# Catheter‐Directed Caudal Epidural Steroid Injection for Chronic Penile Pain With Lumbosacral Radicular Symptoms: A Case Report

**DOI:** 10.1155/cria/6464488

**Published:** 2026-11-02

**Authors:** Sophie E. Smith, Kithmy Wickramasinghe, Moazzam Khan, Alopi Patel

**Affiliations:** ^1^ Department of Anesthesiology and Perioperative Medicine Rutgers-Robert Wood Johnson Medical School New Brunswick New Jersey USA rutgers.edu

## Abstract

Lumbosacral radiculopathy manifesting with pelvic pain symptoms is an underrecognized cause of chronic pelvic pain, as it may be misattributed to primary urologic conditions. In male patients, stigmatization of pelvic pain contributes to underdiagnosis and undertreatment of chronic pelvic pain. This case report discusses a 60‐year‐old male patient with a history of penile and bilateral big toe pain dating back to his teenage years. At the age of 60 years, he was finally diagnosed with lumbosacral radiculopathy and successfully treated with a caudal epidural steroid injection. This case underscores the necessity for physicians to consider lumbosacral radiculopathy as a possible cause for pelvic pain.

## 1. Introduction

Chronic pelvic pain, defined as persistent, noncyclical pain in the pelvic region lasting at least 6 months, is an underdiagnosed and undertreated condition that is estimated to affect between 2% and 16% of males [1, 2]. Undertreatment of male chronic pelvic pain is due to a variety of factors, including the wide range of possible etiologies, variability in presenting symptoms, ambiguity about which specialty would be best equipped to treat the patient depending on the underlying cause, and stigma leading to underreporting of symptoms [1].

Chronic pelvic pain in male patients may be genitourinary, gastrointestinal, musculoskeletal, neurologic, vascular, or dermatologic in nature [1]. Lumbosacral radiculopathy manifesting with pelvic pain symptoms is an underrecognized cause of chronic pelvic pain, as it may be misattributed to primary urologic conditions [3, 4]. Radicular pain is caused by compression, irritation, or inflammation of a nerve root, ultimately leading to pain or numbness along the distribution that the affected nerve innervates [5]. The sacral dermatomes may cause pelvic pain in varying areas of the pelvis.

Epidural steroid injections are an option for the treatment of lumbosacral radiculopathy that is not responsive to conservative treatment measures such as physical therapy or oral medication [5]. The goal of an epidural steroid injection is to introduce corticosteroids and local anesthetics into the epidural space to provide a reduction in pain and inflammation along the nerve roots [5]. Caudal epidural steroid injections target the most inferior portion of the epidural space, and this approach may be accessible even in patients with complex anatomical considerations [5, 6].

Although there is evidence for caudal epidural steroid injections for the treatment of lumbosacral spinal conditions, there has been no emphasis in published literature on the use of caudal epidural steroid injections for the treatment of penile pain. Here, we present a case where a caudal epidural steroid injection was successfully used to relieve longstanding penile pain in a male patient.

## 2. Case

A 60‐year‐old male presented to our pain clinic with chronic penile pain characterized by a burning sensation at the glans penis, associated with similar burning and tingling sensations at the tips of both great toes. His medical history was notable for lifelong chronic lower back pain and a penile neuroma that had been surgically removed 28 years prior. The patient has experienced lifelong penile and big toe pain, but the associated burning sensation began approximately 2 years prior to presentation. The pain typically started in the morning and worsened throughout the day. The patient had temporary relief with lumbar extension. His symptoms included numbness and tingling in a distribution consistent with the L5–S1 dermatomes as well as the tip of the penis. He did not have pain with urination or defecation but reported persistent burning sensations during sexual activity. Neurologic examination demonstrated 5/5 strength in the lumbar dermatomes (L2‐S1). Reflexes were 2+ diffusely, and the straight leg test was positive. Sensation, including saddle sensation, and motor function were diffusely intact, and no other deficits were noted. Prior treatments included gabapentin (100 mg three times daily), nonsteroidal anti‐inflammatory drugs, acetaminophen, muscle relaxants, ice, heat, and more than six weeks of physical therapy and home exercise therapy, all without significant relief.

MRI spine in January 2024 showed bilateral spondylolysis with Grade 2 anterolisthesis of L5 on S1, no compression fracture, degenerative changes, and no abnormal enhancement with contrast. The patient underwent L3–L5 spinal fusion in September 2024, which provided partial but incomplete relief of symptoms. He continued to experience numbness and burning at the penis and bilateral great toes, as well as tenderness and tightness in the perineal region consistent with involvement of the transverse perineal musculature. Because of persistent symptoms despite conservative therapy and surgery, further evaluation including electromyography was planned.

A caudal epidural steroid injection with catheter‐directed spread to the sacral nerve roots was performed under fluoroscopic guidance.

At follow‐up 2 weeks later, the patient reported greater than 50% improvement in symptoms, with diminished intensity of the burning pain. By 4 weeks postprocedure, he reported approximately 80% overall pain relief and significant functional improvement. He had no new bowel or bladder dysfunction, weakness, gait disturbances, or changes in sensation.

### 2.1. Procedure Description

Using anteroposterior and lateral fluoroscopic guidance, the sacral hiatus was identified. 1 mL of preservative‐free 1% lidocaine was injected with a 25‐gauge 1.5‐inch needle to create a skin wheal. Through this wheal, a 17‐gauge Tuohy needle was incrementally advanced to contact the sacral hiatus under intermittent fluoroscopy and then guided through the sacrococcygeal ligament into the caudal epidural space. Following negative aspiration for blood or cerebrospinal fluid, contrast was injected to confirm epidural spread under fluoroscopy (Figure 1). A 19‐gauge epidural catheter was then introduced and advanced to the S1–S2 lateral recess. An additional 0.5 mL of contrast was injected, demonstrating appropriate spread along the corresponding nerve root without vascular uptake. The injectate was then administered.

**FIGURE 1:**
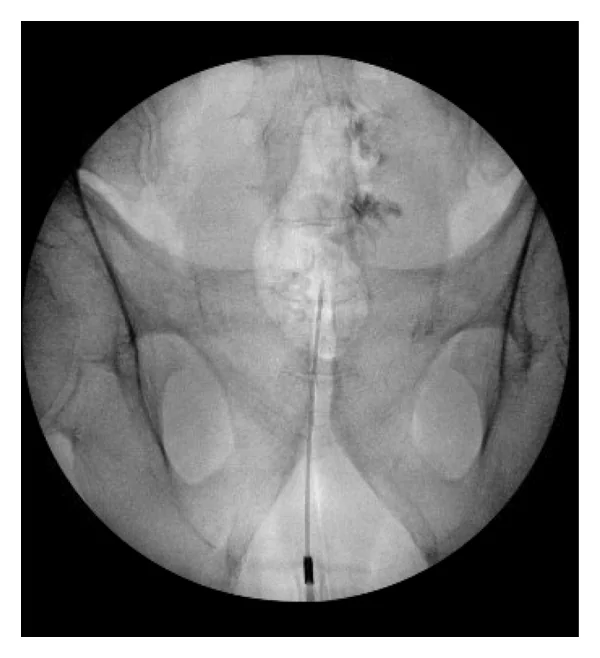
AP view of the sacrum for caudal epidural injection.

## 3. Discussion

Caudal epidural steroid injections have primarily been studied in the context of lower back pain and lower extremity pain, with good evidence for their use to relieve chronic back and lower extremity pain due to disc herniation or radiculopathy [6]. Pelvic pain is a less commonly reported manifestation of radicular pain, but it may occur in the descending nerve distributions affected by lumbosacral radiculopathy and present with pain during micturition, defecation, and orgasm [4].

The neuroanatomic basis for genitopelvic pain is relevant to this case. Somatic sensation of the penis is primarily conveyed through the pudendal nerve, which arises from the S2–S4 nerve roots. The dorsal nerve of the penis, a terminal branch of the pudendal nerve, provides sensory innervation to the penile shaft and glans. Pathology affecting the sacral nerve roots or proximal pathways may manifest as genital pain, even in the absence of a primary penile disorder.

Lumbosacral nerve‐root pathology may, therefore, represent an underrecognized contributor to male pelvic pain. Previous reports have described genital and penile pain associated with spinal pathology. Goldstein et al. demonstrated that pain perceived in the penis can originate from irritated sacral roots or pelvic visceral afferents, even when the penile skin and pudendal nerve are not the primary source [7]. Yan et al. described a case of testicular pain in association with lumbar pathology, further demonstrating that genital pain may arise from peripheral or proximal neural structures rather than from the genitourinary organs themselves [8]. These reports support consideration of spinal and neurogenic etiologies when evaluating patients with otherwise unexplained chronic penile pain.

In this patient, the simultaneous presence of penile burning and bilateral great‐toe paresthesia in the setting of longstanding low back pain raised concern for a lumbosacral neuropathic component. The distribution of symptoms is notable because penile sensation is primarily associated with S2–S4 through the pudendal nerve, whereas the great toe is predominantly associated with the L5 distribution. The involvement of both regions, therefore, does not correspond to a single classic dermatome and may reflect involvement of multiple lumbosacral nerve roots. This distribution also provides a rationale for using a catheter‐directed caudal epidural approach rather than attempting to treat the penile pain as an isolated peripheral pudendal neuropathy. The S2–S4 distribution was targeted as the patient’s penile pain was the most bothersome, and a high‐volume injectate ensured that the medication would reach upper nerve roots beyond S2. In this case, the catheter was advanced toward the S1–S2 lateral recess, allowing delivery of medication within the lower lumbosacral epidural space and potentially exposing multiple adjacent nerve roots. The patient’s response to treatment supports a possible spinal or nerve‐root contribution to his symptoms, although a therapeutic response alone cannot establish causality.

The mechanism by which epidural corticosteroids alleviate radicular pain is thought to involve reduction of inflammation surrounding irritated nerve roots, including suppression of inflammatory mediators and reduction of local edema. The caudal approach may be particularly useful when pathology involves multiple lower lumbar or sacral levels or when prior spinal surgery has altered the anatomy. In this case, catheter‐directed delivery was used to facilitate targeted spread within the epidural space rather than directly injecting the steroid into the penis or pudendal nerve.

While the stigmatization of male pelvic pain is not well‐characterized in the literature, this patient’s difficulty with getting a timely diagnosis and appropriate treatment provides an example of how difficult this process can be for male pelvic pain patients. Although this patient experienced pain symptoms since his late teenage years and mentioned them to doctors many times over the years, his symptoms were dismissed and not taken seriously. This dismissal made him hesitant to continue to seek help for his pain, and the result was that he did not achieve proper diagnosis and treatment for his chronic pain until the age of 60 years.

We urge pain medicine physicians to consider a broad differential when treating their male patients with pelvic pain. Neurogenic pain contributing to pelvic pain in male patients is most commonly misattributed to primary urologic disease [3, 4]. In reality, the possible etiologies of male pelvic pain are much broader and also include gastrointestinal, musculoskeletal, neurologic, vascular, or dermatologic causes in addition to genitourinary causes. More research is needed to better characterize lumbosacral radiculopathy as a cause of pelvic pain.

## 4. Conclusion

After receiving caudal epidural steroid injections, this patient experienced significant relief of his penile and big toe pain. This patient faced stigma, diagnostic challenges, and years of chronic pain before he was diagnosed with a spinal pathology. His clinical course illustrates that chronic penile pain may have a neurogenic or spinal component, particularly when accompanied by lower extremity paresthesia and a history of lumbar pathology. The response to caudal epidural treatment suggests that lower lumbosacral nerve‐root pathology may have contributed to his symptoms, although further studies are needed to establish efficacy. This case highlights the need for additional research and education to evaluate the use of caudal epidural steroid injections to treat pelvic pain due to lumbosacral radiculopathy.

## Funding

No funding was received for this manuscript.

## Conflicts of Interest

The authors declare no conflicts of interest.

## Data Availability

Data sharing is not applicable to this article as no datasets were generated or analyzed during the current study.
